# Space‐Confined Growth of Ultrathin 2D β‐Ga_2_O_3_ Nanoflakes for Artificial Neuromorphic Application

**DOI:** 10.1002/smsc.202400241

**Published:** 2024-09-12

**Authors:** Mingli Liu, Shuai Liu, Jian Yao, Yu Teng, Lin Geng, Alei Li, Lin Wang, Yunfei Li, Qing Guo, Zongjie Shen, Lixing Kang, Mingsheng Long

**Affiliations:** ^1^ Information Materials and Intelligent Sensing Laboratory of Anhui Province Key Laboratory of Structure and Functional Regulation of Hybrid Materials of Ministry of Education Institutes of Physical Science and Information Technology Anhui University 111 Jiu Long Road Hefei 230601 China; ^2^ Advanced Materials Division Suzhou Institute of Nano‐Tech and Nano‐Bionics Chinese Academy of Sciences 398 Ruoshui Road Suzhou 215123 China

**Keywords:** 2D metal oxides, artificial synapses, space‐confined growths, β‐Ga_2_O_3_ nanoflakes

## Abstract

In recent years, wide‐bandgap semiconductor β‐Ga_2_O_3_ material has been widely studied because of its excellent properties. Simultaneously, 2D metal oxides (2DMOs) have also become a focus of research owing to their superior stability and unique physical properties arising from quantum confinement effects. Therefore, the exploration of 2D β‐Ga_2_O_3_ is expected to reveal its novel electrical properties in electronic applications. However, the synthesis of high‐quality 2D β‐Ga_2_O_3_ remains a formidable challenge. Herein, a confined space is constructed to synthesize high‐quality 2D β‐Ga_2_O_3_ nanoflakes by enhancing the control of the kinetics of chemical vapor deposition process. In the device results, it is shown that the grown nanoflakes have excellent switching properties and potential artificial synaptic response characteristics. Based on this premise, an artificial recognition system for handwritten numerals is developed, achieving a peak recognition accuracy of approximately 96%. This system holds significant potential for application within an emerging neuromorphic recognition framework tailored for advanced driver‐assistance systems. In this work, a new feasible pathway is provided for the synthesis of 2D non‐layered oxides and the potential of 2D oxides in the field of neuroanalog electronics and recognition is shown, thereby advancing the fields of 2D β‐Ga_2_O_3_ electronics and 2DMOs electronics.

## Graphical abstract

β‐Ga_2_O_3_, a wide‐bandgap semiconductor, has gained attention for its excellent properties, particularly in its 2D form. In this work, high‐quality 2D β‐Ga_2_O_3_ nanoflakes is synthesized using space‐confined chemical vapor deposition, achieving excellent electrical switching and a 96% accuracy in handwritten numeral recognition. Also, in this work, the synthesis of 2D non‐layered oxides and their applications in neuromorphic systems is advanced.

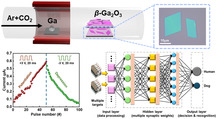

## 1 Introduction

In recent years, wide‐bandgap semiconductor materials have been extensively researched and rapidly developed due to their unique properties, such as high thermal conductivity, high breakdown voltage, and high conduction current.^[^ ^1^, ^2^ ^]^ Among these materials, β‐Ga_2_O_3_ stands out with an indirect bandgap of approximately 4.90 eV, a high breakdown electric field of around 8 mV cm^−1^, and exceptional thermal and chemical stability.^[^ ^3^ ^]^ These remarkable properties of β‐Ga_2_O_3_ have made it a promising material candidate for applications in high‐temperature gas sensors, high‐power field‐effect transistors (FETs), neural mimicry devices, ultraviolet photodetectors, and photonic switches.^[^ ^3^, ^4^ ^]^

Due to the quantum confinement effect, 2D materials exhibit various superior properties compared to their bulk counterparts, including high carrier mobility, tunable bandgap, and enhanced resilience against short‐channel effects.^[^ ^5^, ^6^ ^]^ Therefore, 2D materials are identified as exceptionally promising candidates for next‐generation electronics.^[^ ^6^, ^7^, ^8^, ^9^ ^]^ Particularly, 2D metal oxides (2DMOs) have shown great potential in electronics due to good environmental stability and low synthesis cost.^[^ ^10^, ^11^ ^]^ For instance, the 2D hexagonal TiO_2_ reported by Ou et al. exhibits excellent p‐type semiconductor properties with high hole mobility up to 950 cm^2^ V^−1^ s^−1^.^[^ ^12^ ^]^ Zhai's group achieved a subthreshold swing of 64 mV dec^−1^ in MoS_2_‐based FETs by leveraging 2D Sb_2_O_3_ as the medium.^[^ ^13^, ^14^ ^]^ Furthermore, 2DMOs have exhibited promise in applications such as resistive memory, memory transistors, and electrophoretic memristors, indicating their significant potential for future neuromorphic computing systems.^[^ ^10^ ^]^ Earlier theoretical predictive studies suggest that the emergence of 2D β‐Ga_2_O_3_ could significantly advance gate dielectrics and tunnel barriers within the realm of 2D electronics.^[^ ^15^, ^16^, ^17^, ^18^ ^]^ For example, Hua et al. used first principles to predict that 2D β‐Ga_2_O_3_ has higher carrier mobility and better electronic device performance.^[^ ^18^ ^]^ The excellent properties predicted by these studies motivate researchers to make new attempts in 2D β‐Ga_2_O_3_ synthesis.

Nevertheless, the experimental synthesis of 2D β‐Ga_2_O_3_ encounters certain challenges. While quasi‐2D β‐Ga_2_O_3_ can be obtained through mechanical exfoliation from its bulk crystal, the resulting thickness often exceeds 100 nm due to the constraints imposed by its non‐van der Waals lamellar structure.^[^ ^19^, ^20^ ^]^ Conventional synthesis techniques (radio‐frequency magnetron sputtering,^[^ ^21^, ^22^ ^]^ halide vapor phase epitaxy,^[^ ^23^ ^]^ etc.) have shown progress in producing Ga_2_O_3_ thin films, but these methods typically result in polycrystalline or amorphous films. Alternative approaches, such as liquid metal printing,^[^ ^24^ ^]^ have the capability to generate ultrathin Ga_2_O_3_; however, the amorphous nature of the resulting structure significantly hampers the electrical properties. The synthesis of high‐quality 2D β‐Ga_2_O_3_ single crystals remains a formidable obstacle. Consequently, the development of a novel synthesis method for 2D β‐Ga_2_O_3_ is imperative.

Space‐confined synthesis has demonstrated unique advantages in synthesizing 2D materials such as graphene, metal chalcogenides, 2D perovskites, and 2DMOs, owing to its convenience, efficiency, and universality.^[^ ^25^, ^26^, ^27^, ^28^, ^29^ ^]^ The confined space effectively retards mass transfer dynamics and crystal growth kinetics, thereby enhancing the controllability of 2D material growth processes. Here, we have successfully prepared high‐quality ultrathin 2D β‐Ga_2_O_3_ nanoflakes on a substrate by space‐confined chemical vapor deposition (CVD). The as‐grown 2D β‐Ga_2_O_3_ nanoflakes were comprehensively characterized using optical microscopy (OM), atomic force microscopy (AFM), Raman spectroscopy, X‐ray photoelectron spectroscopy (XPS), and transmission electron microscopy (TEM), revealing their single‐crystalline structure with exceptional crystallization quality. The device results show that the grown nanoflakes have excellent switching properties and potential artificial synaptic response characteristics. Based on the electrical results, the constructed handwriting numbers recognition system achieves an outstanding recognition accuracy of about 96%, and the neuromorphic recognition system (NRS) exhibits promising potential for integration into advanced driver‐assistance systems (ADASs). This study not only establishes an efficient approach for the space‐confined synthesis of 2DMOs but also furnishes insights into the utilization of novel 2DMOs like 2D β‐Ga_2_O_3_ in the realm of electronic devices.

## 2 Results and Discussion

### 2.1 Space‐Confined Growth and Structural Characterization of Ga_2_O_3_ Nanoflakes

**Figure 1:**
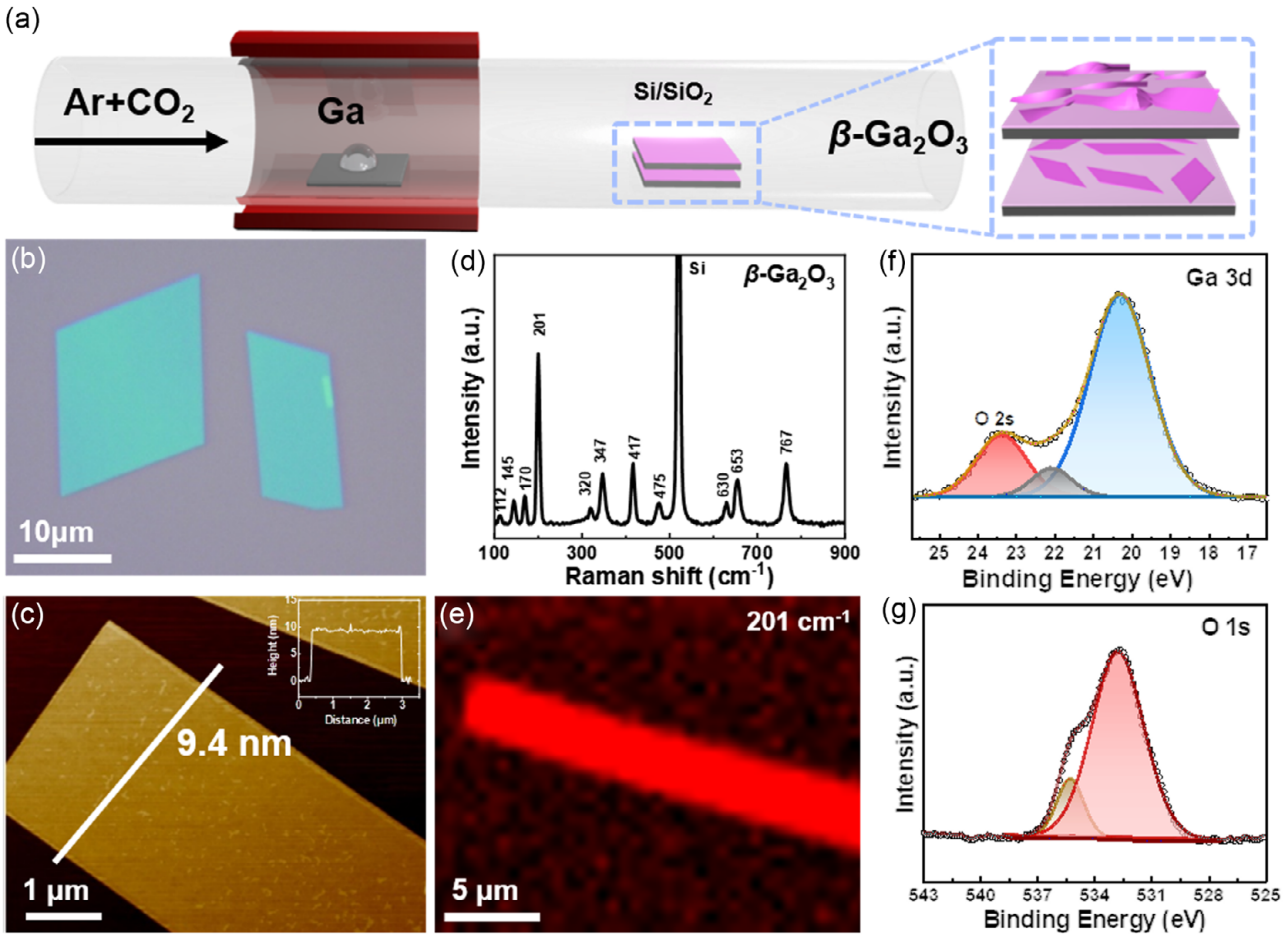
a) Schematic illustration for the space‐confined synthesis of 2D β‐Ga_2_O_3_ nanoflakes. b) OM of 2D β‐Ga_2_O_3_ nanoflakes on SiO_2_/Si substrate. c) AFM topography of β‐Ga_2_O_3_ nanoflakes and corresponding height profile. Raman spectrum of d) β‐Ga_2_O_3_ nanoflakes and the Raman mapping image e) at 201 cm^−1^. XPS of the β‐Ga_2_O_3_ nanoflakes for f) Ga 3*d* and g) O 1*s*.

The growth of 2D β‐Ga_2_O_3_ nanoflakes is performed in a chemical vapor deposition system (**Figure** **1**a). In brief, gallium metal is reacted with an appropriate amount of CO_2_ gas passed at a specific temperature, generating β‐Ga_2_O_3_ downstream. Specifically, we set up a confined space on the substrate to enhance control over gas mass transfer and growth kinetics for achieving the growth of 2D β‐Ga_2_O_3_.^[^ ^27^ ^]^ As reported in the previous article, the gas flow rate is greatly reduced in the confined space constructed by the substrate stack, which means that the precursor transport speed is also greatly reduced, which can effectively reduce the nucleation rate and growth rate.^[^ ^30^, ^31^ ^]^ In this case, the gallium source is transported to the confined space of the substrate with the carrier gas flow and the oxygen source, and the adsorption nucleation growth is carried out on the substrate.^[^ ^32^ ^]^

Details of sample synthesis are provided in Experimental Section. The resulting sample, as shown by OM in Figure 1b, successfully generated regular quadrilateral flakes on SiO_2_/Si substrate, which can reach tens of microns in size. AFM images of the sample show that the thickness of the nanoflakes can be less than 10 nm (Figure 1c), which is very thin for non‐layered materials. However, when a confined space is not constructed, the β‐Ga_2_O_3_ tends to grow into thicker micron bands (Figure S1, Supporting Information). This indicates that the proposed confined space is effective for the growth of non‐layered 2D β‐Ga_2_O_3_.

Raman spectroscopy is a powerful tool for analyzing the structure of 2D materials. Here, the grown samples are characterized by Raman spectroscopy. In the Raman spectrum of Figure 1d, 11 peaks are shown, which appear at 112, 145, 169, 201, 319, 349, 416, 474, 629, 653, and 765 cm^−1^, respectively. These Raman characteristic peaks are in good agreement with the characteristics of mono‐diagonal Ga_2_O_3_,^[^ ^33^, ^34^ ^]^ indicating that the grown material is 2D β‐Ga_2_O_3_. Then, according to the characteristic peak intensity, 201 cm^−1^ was selected to carry out the Raman intensity mapping image of the nanoflake (Figure 1e), and the uniform color distribution showed that the β‐Ga_2_O_3_ nanoflakes have uniform structure. In addition, the crystal structure of the growth samples was further confirmed by X‐ray diffraction (XRD). The XRD pattern of the sample is in good agreement with the standard β‐Ga_2_O_3_ (PDF #43‐1012), which confirms that the obtained nanoflakes are monoclinic Ga_2_O_3_ (Figure S2, Supporting Information). The chemical state of the sample was analyzed by XPS. In the XPS spectrum with energies ranging from 0 to 1200 eV, the signal of the respective energy levels of Ga and oxygen elements can be clearly observed (Figure S3, Supporting Information), and it can be determined that the sample is composed of Ga and O atoms. Among them, the chemical state of Ga 3*d* can be consistent with the previously reported values from the peak of 20.4 eV with binding energy (Figure 1f) and the O 1*s* XPS signal with binding energy of 532.8 eV (Figure 1g), which can correspond to the valence states of the two elements in β‐Ga_2_O_3_.^[^ ^35^, ^36^ ^]^

**Figure 2:**
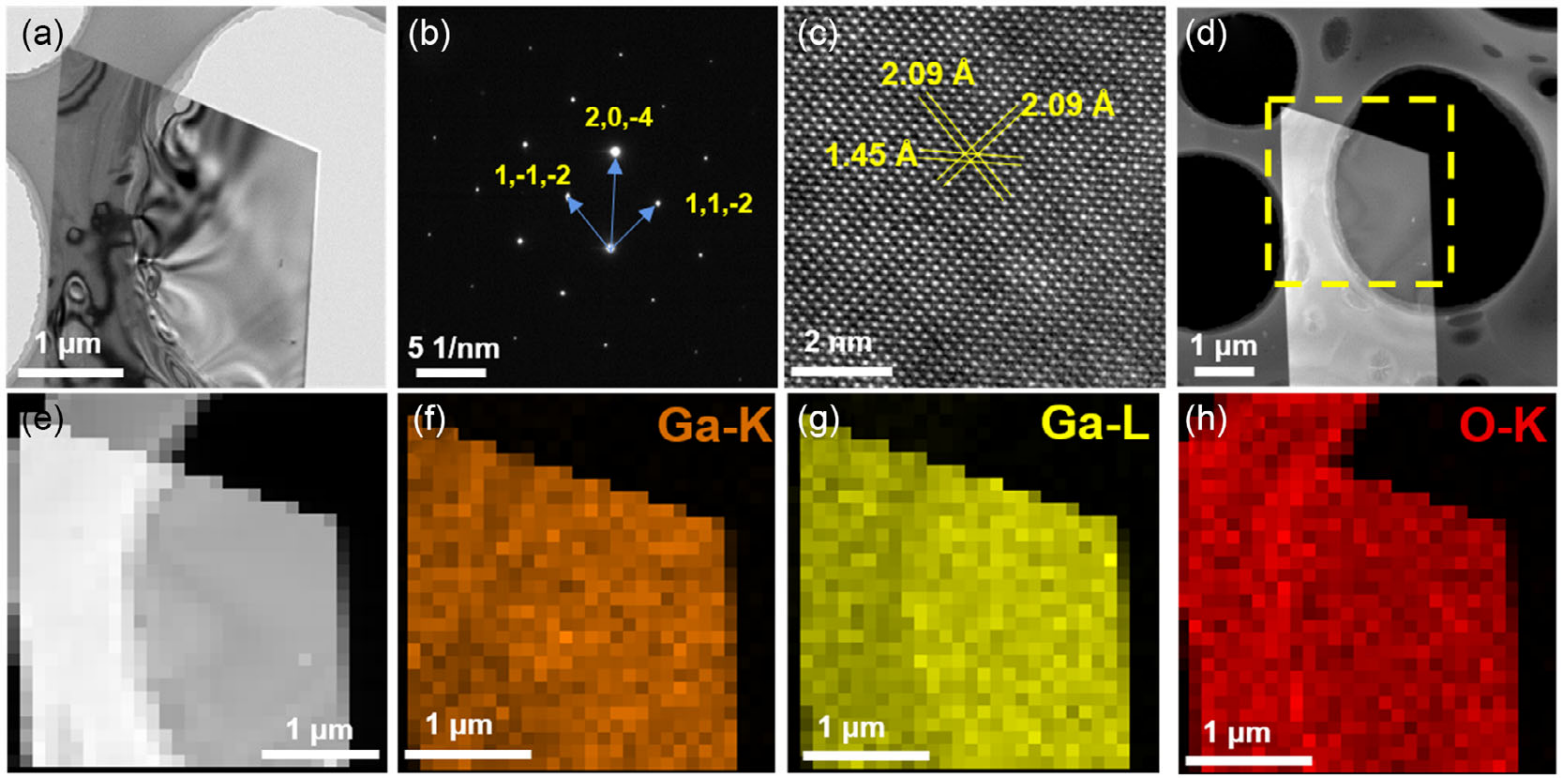
a) TEM image of a quadrilateral β‐Ga_2_O_3_ nanoflakes. b) SAED pattern of the β‐Ga_2_O_3_ nanoflakes. c) HRTEM images of the β‐Ga_2_O_3_ nanoflakes. d–h) HAADF image of β‐Ga_2_O_3_ nanoflake and the corresponding EDX mapping images for Ga and O.

To investigate the crystallinity and quality of the nanoflakes, further structural characterization was performed. The samples of 2D β‐Ga_2_O_3_ were transferred to the Cu grid for TEM characterization. **Figure** **2**a shows a typical 2D β‐Ga_2_O_3_ nanoflake, and the selected area electron diffraction (SAED) was performed (Figure 2b). There is a set of parallelogram diffraction points in the SAED image, which confirms the excellent single‐crystal structure of the nanoflake. Combined with the high‐resolution TEM (HRTEM) image in Figure 2c and the standard card (PDF #43‐1012), the zone axis of the sample was obtained [201]. The high‐angle annular dark‐field scanning TEM (HAADF–STEM) image and the energy‐dispersive X‐ray (EDX) spectroscopy mappings show that Ga and O are evenly distributed (Figure 2e–h). The elemental composition of 2D β‐Ga_2_O_3_ nanoflakes was analyzed in the EDX spectroscopy (Figure S5, Supporting Information). These results confirm the successful preparation of ultrathin 2D β‐Ga_2_O_3_ single crystal, which provides a good material basis for further electronic research and application.

### 2.2 Electronic Properties of 2D β‐Ga_2_O_3_ Nanoflakes

In recent years, 2D materials have become increasingly popular in the fields of semiconductor and micro–nano fabrication. The research on artificial neuromorphic devices has been considered one of the most representative of the effective integration of semiconductors and micro–nano fabrication technologies. The 2D semiconductor materials have received widespread attention in the neuromorphic device field due to their advantages, such as atomic‐level thickness, the surface without dangling bonds, low power consumption, and high electrical conductivity.^[^ ^37^, ^38^, ^39^, ^40^, ^41^, ^42^, ^43^, ^44^ ^]^ We hereby provided a brief comparison of some mainstream 2D semiconductor channel materials to discuss the practical implementation of artificial neuromorphic applications, including synthesis methods of channel materials and electronic properties of fabricated devices. Xie et al. proposed a neuromorphic computing system with 2D MoS_2_ memtransistors. A facile coplanar multigate memtransistor based on a 2D MoS_2_ channel layer and a poly(vinyl alcohol) dielectric gate mimicked the performance of spatiotemporally processed visual neurons.^[^ ^45^ ^]^ Reham et al. demonstrated an analog‐digital hybrid computing platform based on a 2D SnS_2_ memtransistor for energy‐efficient and reconfigurable sensor fusion with a complementary filter algorithm. The memtransistor with the SnS_2_ channel exhibited multiple conductance states, which could be modulated by minimal energy consumption.^[^ ^39^ ^]^ Gong et al. reported an artificial vision system integrating the processing capability of visual information sensing memory. The system operated with a 6 × 6 memtransistor array, and the 2D WSe_2_ material played the role of semiconductor channel. By modulating carrier types of the WSe_2_ channel with different thicknesses, highly linear symmetric plasticity was obtained to facilitate the high level of training and inference accuracy for artificial neural networks (ANN).^[^ ^46^ ^]^

**Figure 3:**
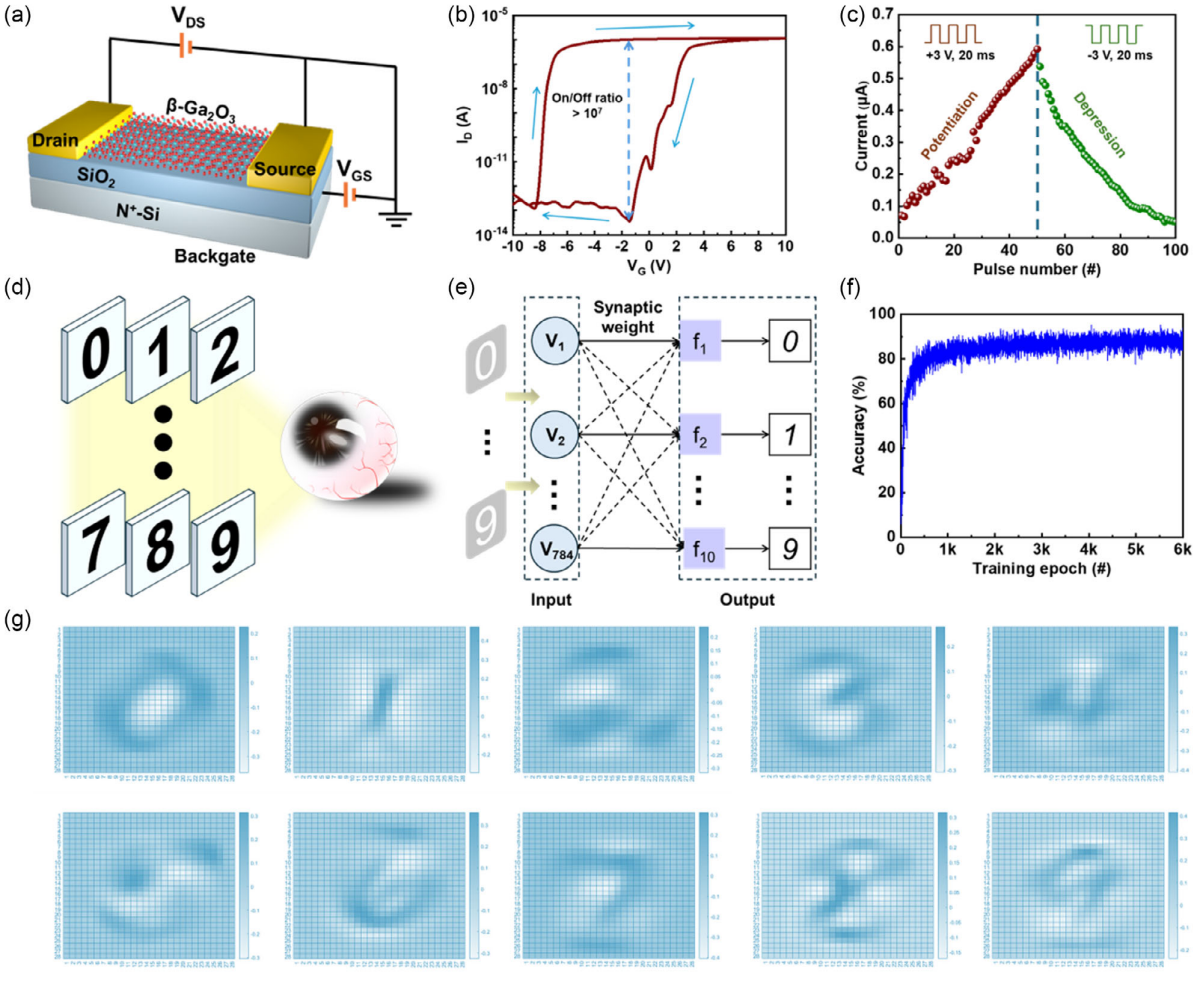
a) Schematic view of the memtransistor with the 2D β‐Ga_2_O_3_ channel. b) The transfer characteristic of the β‐Ga_2_O_3_ memtransistor. c) Neuromorphic performance with long‐term potentiation and depression responses of the β‐Ga_2_O_3_ memtransistor. d) Schematic view of artificial recognition for handwriting numbers. e) Schematic diagram of the MLP ANN. f) The statistic results of recognition accuracy for handwriting numbers. g) Output results with handwriting numbers of the artificial recognition system.

Herein, to investigate the potential of this ultrathin 2D β‐Ga_2_O_3_ nanoflakes synthesized by the space‐confined CVD method, a memtransistor device with the 2D β‐Ga_2_O_3_ channel was proposed to explore the artificial neuromorphic performance. Some other 2D semiconductor materials were also investigated, and related comparison results can be observed in Table S1, Supporting Information. The back‐gate structure memtransistor with the 2D β‐Ga_2_O_3_ semiconductor channel and SiO_2_ dielectric can be observed in **Figure** **3**a. As illustrated in Figure 3b, the transfer characteristic evaluated by the sweeping voltage bias of the memtransistor exhibited a clockwise hysteresis with an On/Off ratio higher than 10^7^, which provided an excellent memory platform for further investigation of artificial synaptic responses.^[^ ^47^, ^48^, ^49^ ^]^ Some supplementary electrical characterizations can be observed in Figure S7–S9, Supporting Information, which include repeated transfer hysteresis, endurance measurement (≈400 cycles), and retention performance (>2 × 10^4^ s). These results indicated the excellent reliability and stability of the 2D β‐Ga_2_O_3_ memtransistor. With consecutive pulse stimulations (±3 V, 20 ms), the typical neuromorphic performance with long‐term potentiation (50 positive pulses) and depression (50 negative pulses) responses of the β‐Ga_2_O_3_ memtransistor can be obtained in Figure 3c, which indicates the application potential of the β‐Ga_2_O_3_ memtransistor in the field of artificial neuromorphic computing.^[^ ^48^, ^49^ ^]^ Therefore, an artificial recognition system for handwriting numbers (Figure 3d) was constructed with the ANN and original data from the Modified National Institute of Standards and Technology (MNIST) data set.^[^ ^50^, ^51^ ^]^ The schematic diagram of ANN in Figure 3e was constructed with classic multiple‐layer perceptron (MLP) layers, including an input layer with 784 neurons, a hidden layer with synaptic weights, and an output layer with 10 neurons. For the whole number recognition system, four parameters from the long‐term potentiation and depression responses should be obtained, including the maximum conductance (*G*_MAX_), the minimum conductance (*G*_MIN_), the nonlinearity of the potentiation curve (*N*_L–P_), and the nonlinearity of the depression curve (*N*_L–D_). According to the fitting results based on the power‐allometric model, these four parameters were 5.047 × 10^−8^, 5.920 × 10^−8^, 0.271, and 0.413.^[^ ^52^, ^53^ ^]^ The final recognition accuracy with statistic results can be observed in Figure 3f. The average value of recognition accuracy was higher than 90%, and the highest could be up to around 96%.

**Figure 4:**
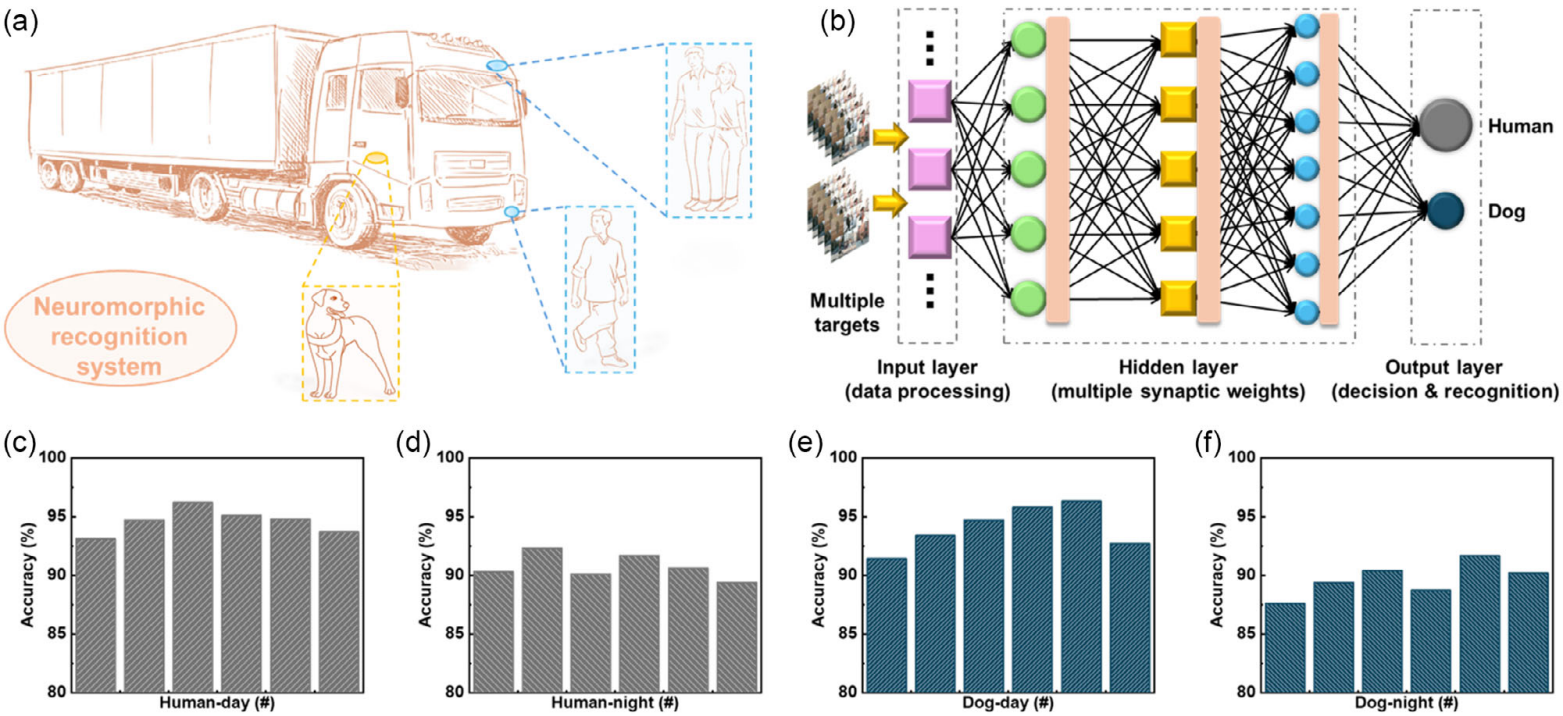
a) Schematic view of the neuromorphic recognition system for the artificial intelligence vehicle. b) Schematic diagram the YOLO‐v5 network with multiple layers. Recognition accuracy of c) human in daytime, d) human in nighttime, e) dogs in daytime, and f) dogs in nighttime.

Based on the recognition results from ANN mentioned earlier, an emerging NRS prepared for the ADAS was constructed with the multilayer You Only Look Once‐version 5 (YOLO‐v5) neuromorphic network. As illustrated in **Figure** **4**a, this NRS was used to simulate the behavior of vehicles recognizing obstacles (such as humans and dogs) on the road during the driving process. The operation foundation of this NRS was the function integration of multiple data acquisition, data analysis, computational interpreting, and output prediction, which was associated with the construction of the neuromorphic networks and algorithms.^[^ ^54^, ^55^, ^56^ ^]^ Multiple properties with variations of desired targets should be under consideration, including size, position, and movement state, which have presented high requirements for recognition efficiency and accuracy.^[^ ^55^, ^56^ ^]^ The structure of the YOLO‐v5 neuromorphic network can be observed in Figure 4b, including the specific functions of each neural layer. Humans and dogs were selected as commonly identified targets with size variation in this recognition process. Related recognition data can be obtained in Figure S10–S13, Supporting Information. Image sets with different brightness and contrast were utilized to simulate recognition environments during the daytime and nighttime, respectively. Figure 4c,d exhibits recognition results of humans in daytime and nighttime, and higher recognition accuracy results (Figure 4c) were obtained in the daytime environment. The average accuracy was around 93%, and the highest accuracy could be up to 97%. Relative‐worse recognition results for humans were obtained at night (Figure 4d) owing to the reduction of brightness and contrast. Similar results were also observed in the recognition process for dogs, as illustrated in Figure 4e,f.

## 3 Conclusions

In summary, to synthesize high‐quality 2D β‐Ga_2_O_3_ nanoflakes, we constructed a confined space to strengthen the control of the kinetics of the 2D β‐Ga_2_O_3_ chemical vapor deposition process. Therefore, ultrathin 2D β‐Ga_2_O_3_ nanoflakes with high quality were successfully prepared on the substrate. The 2D β‐Ga_2_O_3_ nanoflakes were characterized by OM, AFM, Raman spectroscopy, XPS, and TEM. The results show that the prepared 2D β‐Ga_2_O_3_ nanoflakes have a single‐crystal structure. The electrical test results show that the nanoflakes have excellent switching properties and potential artificial synaptic response characteristics. On this basis, the recognition accuracy of the handwritten digit recognition system can be as high as 96%, and the NRS shows good potential for integration with ADASs. This work provides a new feasible approach for the synthesis of 2D non‐layered oxides and shows the potential of 2DMOs in the field of neural analog electronics and recognition, thus promoting the development of Ga_2_O_3_ electronics and 2DMOs electronics.

## 4 Experimental Section

4.1

4.1.1

##### Synthesis of 2D β‐Ga_2_O_3_ Flakes

The 2D β‐Ga_2_O_3_ nanoflakes were prepared by atmospheric pressure CVD system. In the growth stage, gallium (Ga 99.999%, Aladdin) is used as the precursor; trace carbon dioxide was added as the oxygen source; and argon (Ar) was used as the carrier gas. About 10 mg of gallium was placed on the supported W foil (50 um thick). The gallium source was then placed in the middle of the quartz tube heating area, and the growing substrate was placed 8 cm away from the gallium source. To construct the restricted space, two silicon substrates about 1 cm by 1 cm were stacked face to face. First, in a mixed atmosphere of Ar and hydrogen (H_2_), the temperature gradually rose to 1000 °C. H_2_ was introduced in part to ensure that gallium was not oxidized before the target temperature was reached. Carbon dioxide (5–30 sccm) was then pumped in to replace the hydrogen and initiate growth. Finally, after 5–15 min of growth, 2D β‐Ga_2_O_3_ flakes could be obtained on the substrate.

##### Characterizations of 2D β‐Ga2O3 Nanoflakes

The 2D β‐Ga_2_O_3_ nanoflakes were further characterized by OM (VK‐X260K), AFM (Dimension Icon, with a tapping mode), scanning electron microscope (S‐4800, HITACHI), Raman (HORIBA, with a 532 nm laser as excitation source), XRD (D8 Advance), and XPS (PHI 5000 VersaProbe II). The HRTEM and SAED measurements were performed on a TEM (Tecnai G2 F20 S‐TWIN, FEI). Samples for TEM testing were obtained by transferring thin sheets of 2D β‐Ga_2_O_3_ grown on SiO_2_/Si to a TEM copper grid by PMMA.

##### Device Fabrication and Electrical Measurement

The memtransistor was presented with a SiO_2_ dielectric layer and a 2D β‐Ga_2_O_3_ channel. To remove impurities on the surface, the SiO_2_/Si substrate was ultrasonically cleaned in acetone, ethyl alcohol, and deionized water, respectively. Then, the 2D β‐Ga_2_O_3_ nanoflakes were deposited on the SiO_2_/Si. Finally, Ti/Au (5/40 nm) was deposited as source and drain electrodes. All electrical characterizations were measured by the Agilent 1500 semiconductor analyzer.

## Conflict of Interest

The authors declare no conflict of interest.

## Author Contributions

**Mingli Liu**: Conceptualization (equal); Investigation (equal); Writing—original draft (lead). **Shuai Liu**: Conceptualization (equal); Investigation (equal); Writing—original draft (lead). **Jian Yao**: Formal analysis (equal); Methodology (supporting). **Yu Teng**: Formal analysis (supporting); Methodology (equal). **Lin Geng**: Investigation (supporting). **Alei Li**: Formal analysis (supporting); Investigation (supporting); Methodology (supporting). **Lin Wang**: Investigation (supporting). **Yunfei Li**: Investigation (supporting). **Qing Guo**: Conceptualization (supporting); Investigation (supporting); Writing—review & editing (equal). **Zongjie Shen**: Formal analysis (lead); Methodology (lead); Writing—original draft (supporting); Writing—review & editing (supporting). **Lixing Kang**: Conceptualization (lead); Writing—review & editing (lead). **Mingsheng Long**: Conceptualization (lead); Writing—review & editing (lead).

## Supporting information

Supplementary Material

## Data Availability

The data that support the findings of this study are available from the corresponding author upon reasonable request.
