# Supplementary material for: Space‐Confined Growth of Ultrathin 2D β‐Ga2O3 Nanoflakes for Artificial Neuromorphic Application
Source: Small Sci. 2024 Sep 12;4(11):2400241. doi: 10.1002/smsc.202400241 (PMC11935089; doi:10.1002/smsc.202400241)
Supplement: Supplementary file 1 — Supplementary Material [file SMSC-4-2400241-s001.pdf]

Supporting Information

Space-Confined Growth of Ultrathin 2D *β*-Ga_2O_3 Nanoflakes for Artificial Neuromorphic Application

Mingli Liu†, Shuai Liu†, Jian Yao, Yu Teng, Lin Geng, Alei Li, Lin Wang, Yunfei Li, Qing Guo, Zongjie Shen*, Lixing Kang*, and Mingsheng Long**

M. Liu, M. Long

Information Materials and Intelligent Sensing Laboratory of Anhui Province, Key Laboratory of Structure and Functional Regulation of Hybrid Materials of Ministry of Education, Institutes of Physical Science and Information Technology, Anhui University, 111 Jiu Long Road, Hefei, 230601 China

E-mail: longms@ahu.edu.cn

S. Liu, J. Yao, Y. Teng, L. Geng, A. Li, L. Wang, Y. Li, Q. Guo, Z. Shen, L. Kang

Advanced Materials Division, Suzhou Institute of Nano-Tech and Nano-Bionics, Chinese Academy of Sciences, 398 Ruoshui Road, Suzhou, 215123, China.

E-mail: qguo2022@sinano.ac.cn, zjshen2022@sinano.ac.cn, lxkang2013@sinano.ac.cn.

† These authors contributed equally to this work

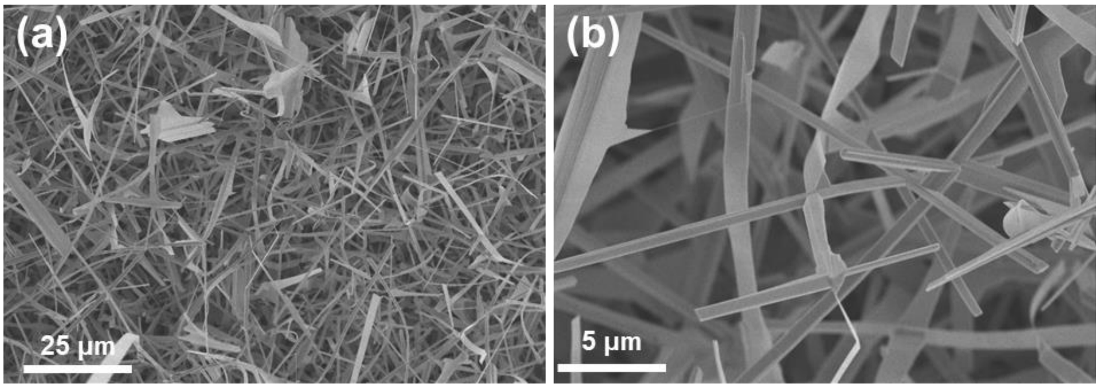

Figure S1. SEM images of β-Ga_2O_3 samples grown freely.

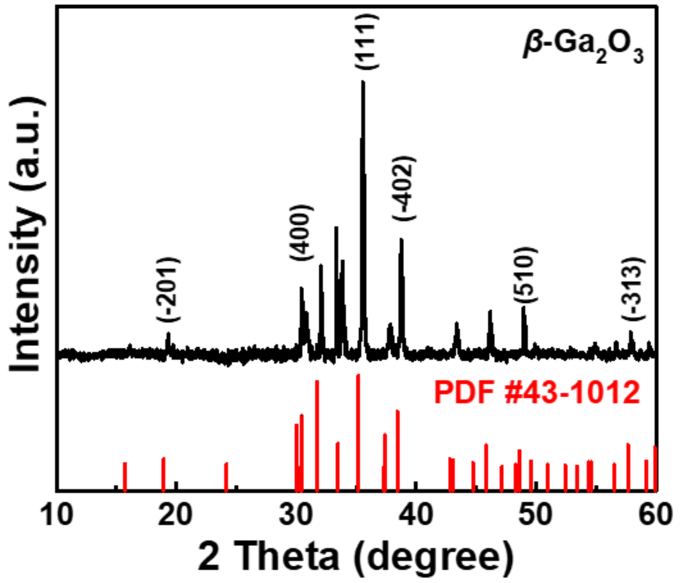

Figure S2. X-Ray Diffraction patterns of β-Ga_2O_3 nanoflakes.

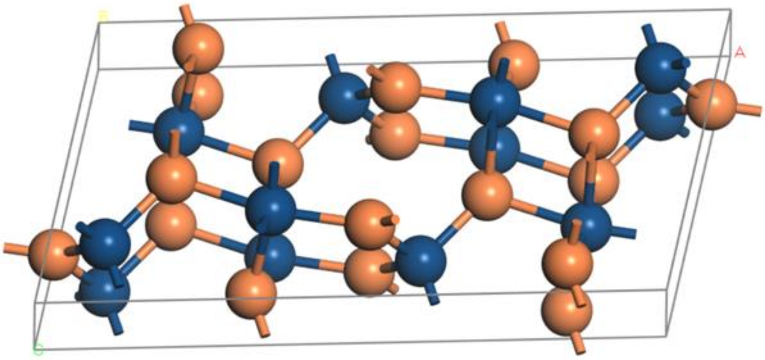

Figure S3. Schematic diagram of the crystal structure of β-Ga_2O_3 nanoflakes.

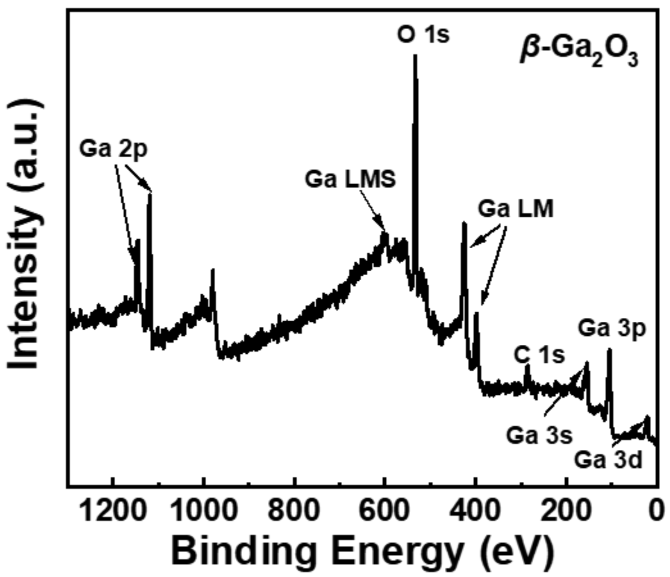

Figure S4. X-ray photoelectron spectroscopy of β-Ga_2O_3 nanoflakes.

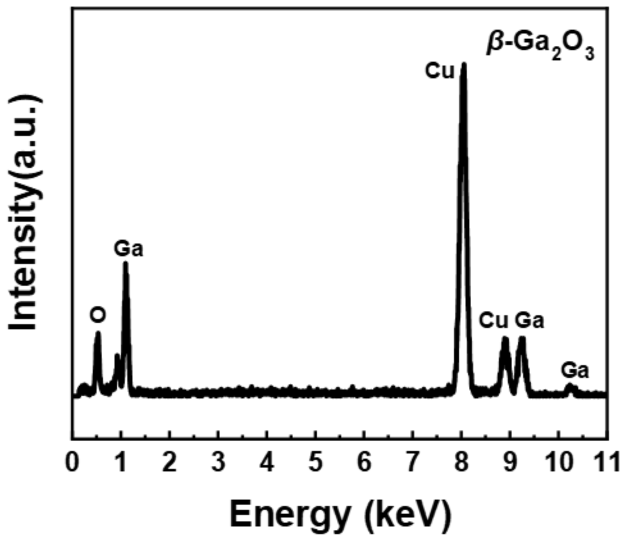

Figure S5. Energy dispersive X-ray spectroscopy of 2D β-Ga_2O_3.

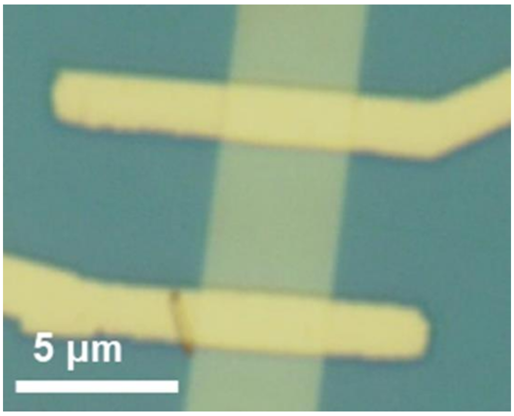

Figure S6. The optical microscope image of 2D β-Ga_2O_3 devices.

Table S1. Comparison of some mainstream 2D semiconductor channel materials

Channel materials	Synthesis methods	Operation voltage	On/Off ratio	Retention time	Endurance cycles	Ref.
MoS_2	CVD	20 V	> 10^2	N. A.	N. A.	[1]
MoS_2	CVD	10 V	> 10^5	3 × 10^4 s	10^4	[2]
SnS_2	CVD	10 V	> 5 × 10^3	N. A.	6 × 10^2	[3]
WSe_2	CVD	8 V	> 10^5	10^4 s	10^2	[4]
$MoSe_2$	CVD	8 V	> 10^4	10^4 s	4 × 10^2	[5]
SnO_2	LMP	15 V	> 10	10^4 s	10^4	[6]
BP	SP	6 V	> 10^2	10^4 s	10^3	[7]
**β-Ga_2O_3**	**CVD**	**10 V**	**> 10^7**	**2 × 10^4 s**	**4 × 10^2**	**Our work**

BP: black phosphorus

CVD: chemical vapor deposition

LMP: liquid-metal printing

SP: solution-processed

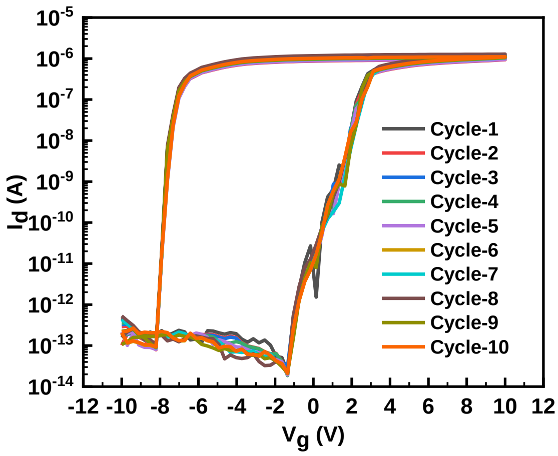

Figure S7. Cycle-to-cycle performance of the 2D β-Ga_2O_3 memtransistor.

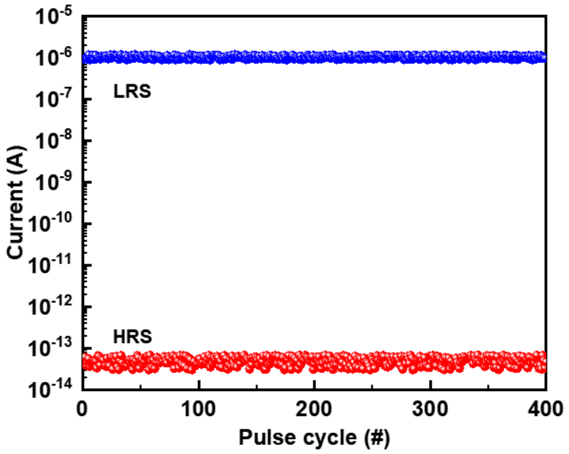

Figure S8. Endurance measurement of the 2D β-Ga_2O_3 memtransistor.

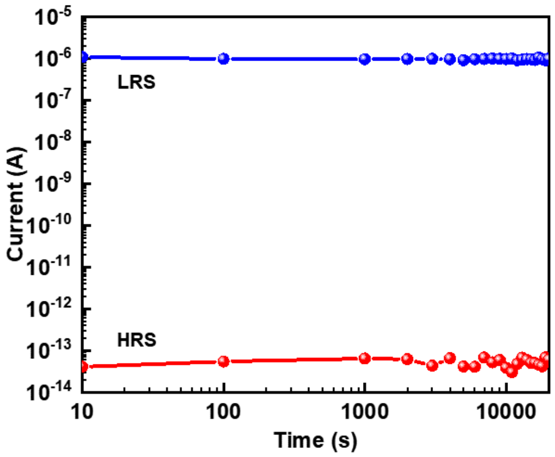

Figure S9. Retention performance of the 2D β-Ga_2O_3 memtransistor.

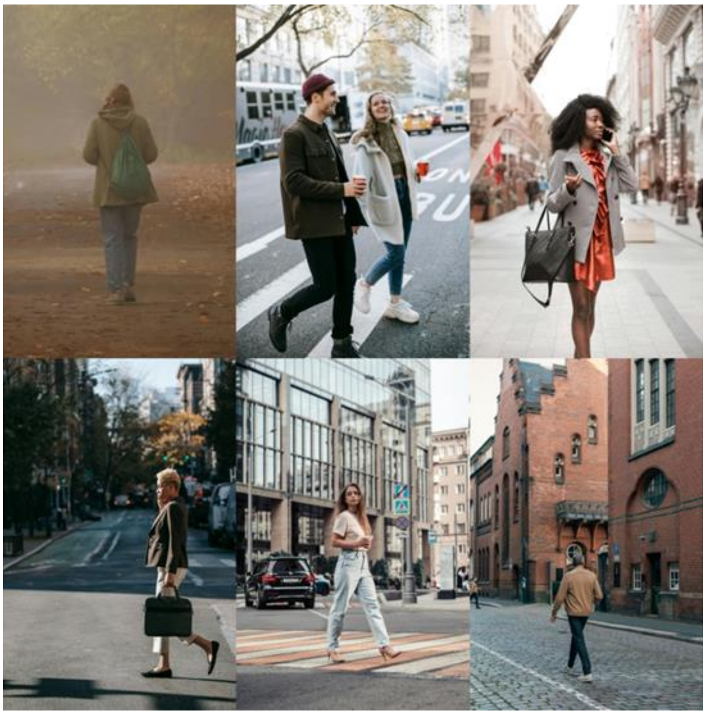

Figure S10. Neuromorphic recognition targets with pedestrians in the daytime.

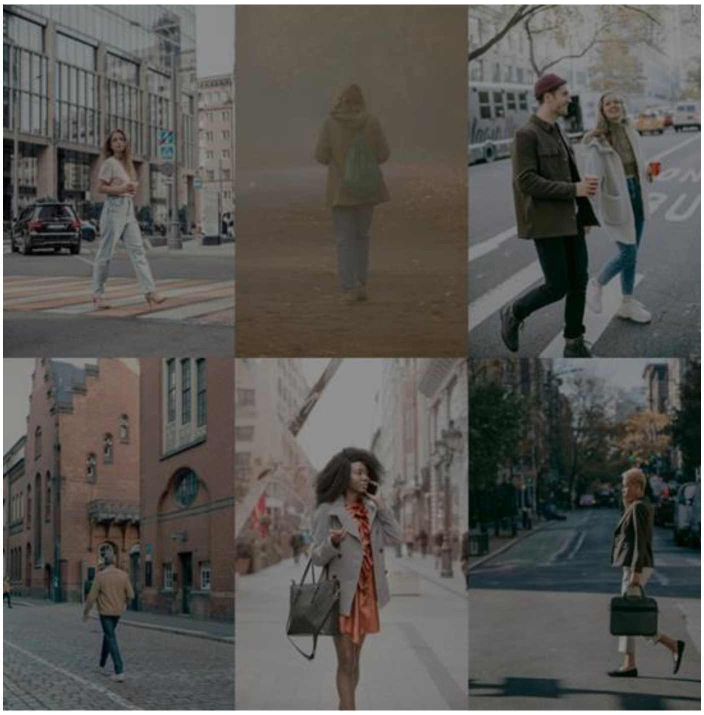

Figure S11. Neuromorphic recognition targets with pedestrians in the nighttime.

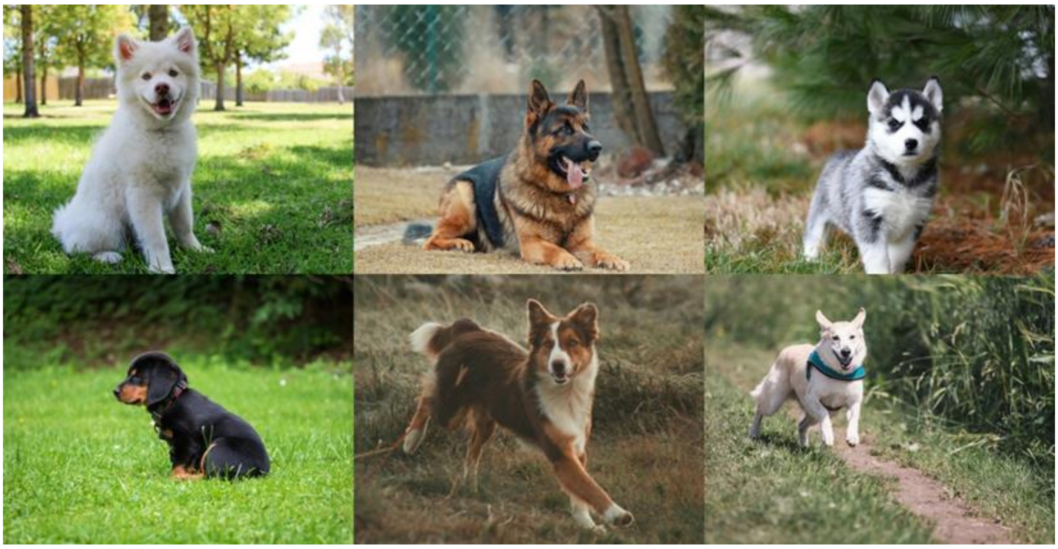

Figure S12. Neuromorphic recognition targets with dogs in the daytime.

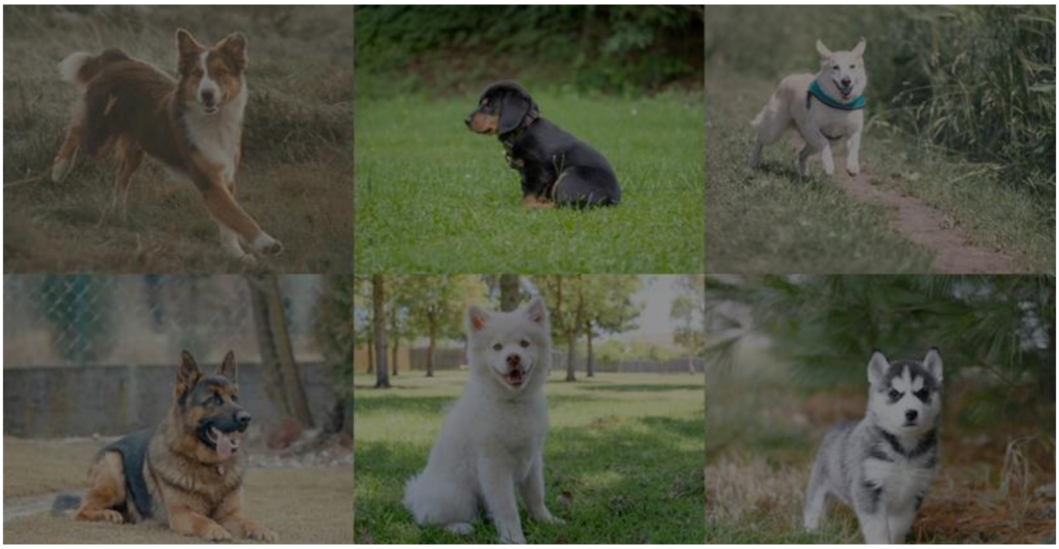

Figure S13. Neuromorphic recognition targets with dogs in the nighttime.

References:

[1] D. Xie, J. Jiang, W. Hu, Y. He, J. Yang, J. He, Y. Gao, Q. Wan, *ACS Appl. Mater. Interfaces* **2018**, *10*, 25943.

[2] M. D. Tran, H. Kim, J. S. Kim, M. H. Doan, T. K. Chau, Q. A. Vu, J. Kim, Y. H. Lee, *Adv. Mater.* **2019**, *31*, 1807075.

[3] S. Rehman, M. F. Khan, H.-D. Kim, S. Kim, *Nano Energy* **2023**, *109*, 108333.

[4] Y. Gong, P. Xie, X. Xing, Z. Lv, T. Xie, S. Zhu, H. Hsu, Y. Zhou, S. Han, *Adv. Funct. Mater.* **2023**, *33*, 2303539.

[5] Y. Jeong, H. J. Lee, J. Park, S. Lee, H.-J. Jin, S. Park, H. Cho, S. Hong, T. Kim, K. Kim, S. Choi, S. Im, *npj 2D Mater Appl* **2022**, *6*, 23.

[6] C.-H. Huang, H. Chang, T.-Y. Yang, Y.-C. Wang, Y.-L. Chueh, K. Nomura, *ACS Appl. Mater. Interfaces* **2021**, *13*, 52822.

[7] D. Kumar, H. Li, U. K. Das, A. M. Syed, N. El-Atab, *Adv. Mater.* **2023**, *35*, 2300446.
